# Functional Activities and Mechanisms of *Aronia melanocarpa* in Our Health

**DOI:** 10.3390/cimb46080477

**Published:** 2024-07-26

**Authors:** Min Young Go, Jinsick Kim, Chae Young Jeon, Dong Wook Shin

**Affiliations:** Research Institute for Biomedical and Health Science, Konkuk University, Chungju 27478, Republic of Korea; rhalsdud1011@kku.ac.kr (M.Y.G.); jindoli477@kku.ac.kr (J.K.); young4mam@kku.ac.kr (C.Y.J.)

**Keywords:** *Aronia melanocarpa*, chokeberry, human, health

## Abstract

*Aronia melanocarpa*, known as black chokeberry, is rich in polyphenols, comprising flavonoids, such as anthocyanins, flavanols, and flavonols, and phenolic acids, such as chlorogenic acid. These polyphenols endow *Aronia melanocarpa* with preventive and therapeutic properties against various human diseases. *Aronia melanocarpa* has beneficial effects against diseases such as diabetes, inflammation, and hypertension. Considering the diverse functional components of *Aronia melanocarpa*, its efficacy in disease prevention and treatment can operate through multiple pathways, offering a more robust approach to disease control. This review covers the latest research results on the functional components of *Aronia melanocarpa* and their effects on human diseases.

## 1. Introduction

*Aronia melanocarpa*, scientifically known as black chokeberry, belongs to the Rosaceae family and hails from the eastern regions of North America. Native Americans highly value *Aronia melanocarpa*, used to brew tea to treat colds and its bark as an astringent [1,2]. Due to their sour taste and astringent nature, *Aronia melanocarpa* pomes are seldom consumed fresh. Instead, they have gained popularity for their use in the mass production of juices, jams, wines, liqueurs, and schnapps [3,4]. 

The berries of *Aronia melanocarpa*, commonly known as Aronia berries, exhibit a range of bioactivities that are advantageous for human health. Studies have revealed that Aronia berries are rich in phenolic compounds, comprising procyanidins, anthocyanins, phenolic acids, and their derivatives [5,6,7]. These bioactive compounds account for biological effects associated with Aronia fruits and indicate the potential for identifying valuable therapeutic agents. *Aronia melanocarpa* confers numerous health benefits, including anti-inflammatory, anti-cancer, anti-diabetic, and anti-atherosclerotic properties [5,6,7].

Polyphenols constitute the most important antioxidants in the human diet [8,9]. Among various berry fruits, *Aronia melanocarpa* stands out as one of the richest sources of polyphenols [10]. These polyphenols possess diverse functional properties, potentially aiding disease prevention and treatment. These functionalities primarily originate from the potent antioxidant activity of polyphenols, which scavenge free radicals and play a notable role in relieving certain chronic diseases. The polyphenolic composition of *Aronia melanocarpa* undergoes notable changes throughout fruit development and ripening, with unripe fruits exhibiting the highest total polyphenol content [11]. These polyphenols are classified into two groups: flavonoids and phenolic acids. Flavonoids include anthocyanins, flavanols, and flavonols, whereas phenolic acids predominantly consist of chlorogenic acid and its isomers. Proanthocyanidins were identified as the primary contributors to the antioxidant activity of *Aronia melanocarpa*, considering the individual phenolic content in the fruits [12]. Cyanidins, in combination with various glycosides, are the major anthocyanins in *Aronia melanocarpa* [13]. Analysis of anthocyanin content indicates cyanidin-3-O-arabinoside and cyanidin-3-O-galactoside as the primary anthocyanins [14].

Cyanidin-3-galactoside is a prominent phenolic compound in *Aronia melanocarpa*, correlating with enhanced antioxidant and radical-scavenging properties [14].

Flavonols are primarily composed of quercetin, which is bound to various glycosides. Phenolic acids are less diverse than flavonoids, with chlorogenic and neochlorogenic acids being their primary constituents [15,16,17]. Quercetin and epicatechin, which are minor phenolic compounds, exhibit the highest antioxidant activity [18]. The total phenol, flavonoid, and proanthocyanidin levels in *Aronia melanocarpa* extracts surpass those in blueberry extracts.

**Figure 1:**
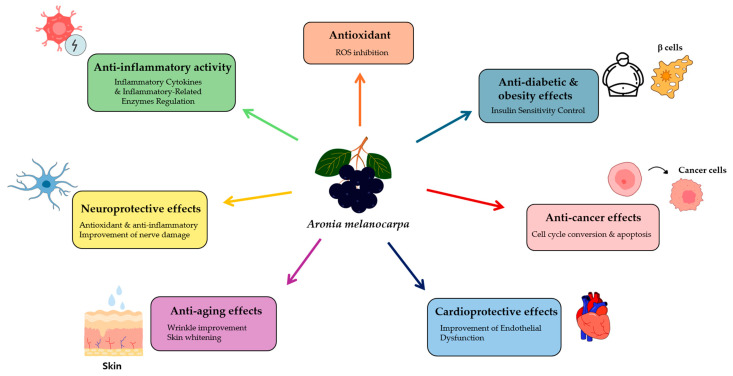
The healthful effects of *Aronia melanocarpa* in various diseases.

This review focuses on the beneficial effects of *Aronia melanocarpa* in various human diseases, including diabetes, heart disease, neuronal disease, and even aging. We also explore the mechanisms of action, molecular targets of the active compounds, and their clinical trial investigations of *Aronia melanocarpa*, highlighting its advantageous health properties (Figure 1).

## 2. Materials and Methods

### 2.1. Search Strategy

Until 24 June 2024, we searched PubMed for published articles that investigated the effects of *Aronia melanocarpa* on health. To reflect the latest research, we limited the study timeframe from 2014 to the present (within ten years). The search combined the keywords “*Aronia melanocarpa*”, “blackberry”, “phenolic acid”, “polyphenol”, “anthocyanin”, “inflammation”, “diabetes”, “heart disease”, “neuronal disease”, and “aging”.

### 2.2. Selection of Studies

Records were chosen by title and/or abstract to exclude studies that did not help answer the question in this review. Inclusion criteria: (1) published in English; (2) intervention included *Aronia melanocarpa*; (3) cell signaling.

## 3. Beneficial Health Effects of *Aronia melanocarpa*

### 3.1. Antioxidant Effects

The leaves of Aronia species possess notable antioxidant capacity, suggesting potential therapeutic and dietary benefits [19]. The extracts and various phenolic compounds derived from Aronia berries demonstrated radical scavenging activity. Additionally, they inhibited 15-lipoxygenase and xanthine oxidase, enzymes known for their peroxidative and prooxidative roles in generating reactive oxygen species (ROS) [19].

Several minor phenolic compounds isolated from Aronia berries exhibit potent antioxidant activity in a hydroxyl radical-scavenging assay. Notably, the presence of the catechol group within these compounds appears to be crucial for mediating such antioxidant properties [20].

Proanthocyanidins are responsible for 40% of *Aronia melanocarpa* in vitro antioxidant activity, followed by anthocyanins (24%), hydroxycinnamic acids (18%), and epicatechins (11%). Procyanidins are superior antioxidants compared to their corresponding monomers [21,22]. Green and unripe *Aronia melanocarpa* exhibit the highest antioxidant activity owing to their elevated levels of procyanidins and flavonoids [23,24]. Cyanidin-3-O-arabinoside exhibits the most potent radical-scavenging properties among the anthocyanins found in *Aronia melanocarpa*, demonstrating strong inhibition of pro-oxidative enzymes [25]. Anthocyanins from black *Aronia melanocarpa*, specifically cyanidin-3-O-galactoside, were isolated. Cyanidin-3-O-galactoside from *Aronia melanocarpa* exhibited significantly higher antioxidant capacity compared to other individual anthocyanins. Furthermore, oral administration of *Aronia melanocarpa* with cyanidin-3-O-glucoside and cyanidin-3-O-galactoside resulted in immunomodulatory effects on the functional activity of phagocytes in vivo [26]. Additionally, another study reported that the daily consumption of 150 mL of juice by rowers engaged in physical exercise during a 1-month training camp reduced exercise-induced oxidative damage to red blood cells [27]. *Aronia melanocarpa* extracts hindered the differentiation of osteoclasts induced by RANKL by diminishing ROS production, deactivating the JNK/ERK/p38 pathways, and the nuclear factor kappa B (NF-κB)-mediated c-Fos and NFATc1 signaling pathways [28].

These findings indicate that *Aronia melanocarpa* extracts are promising sources of natural antioxidants and functional food components (Table 1).

### 3.2. Anti-Inflammatory Activity

The anti-inflammatory properties of *Aronia melanocarpa* fruit are involved in the prevention of chronic diseases, including diabetes, cardiovascular diseases, and immune system disorders [29]. Key pro-inflammatory enzymes, including cyclooxygenases (COXs) and inducible nitric oxide synthase (iNOS), play crucial roles in the synthesis of lipid mediators and nitric oxide (NO), contributing to the progression of numerous inflammatory conditions [30]. *Aronia melanocarpa* extracts showed anti-inflammatory effects on rat endotoxin-induced uveitis [31].

In vitro experiments suggest that the extract’s anti-inflammatory action on ocular inflammation may involve inhibiting NO, prostaglandin, and tumor necrosis factor (TNF) production, achieved through downregulating iNOS and COX-2 enzymes. Extracts inhibit the pro-inflammatory response in human aortic endothelial cells [32]. In addition, a previous study revealed a therapeutic use of *Aronia melanocarpa* bioactive fraction in treating various inflammatory airway disorders. Besides reducing iNOS and COX-2 expression, their study offered clear evidence of anti-inflammatory activity through attenuating ROS secretion and induction of cell cycle arrest [33].

Cyanidin-3-O-galactoside and caffeoylquinic acid inhibited the release of TNF-α, interleukin (IL)-6, and IL-8 in human peripheral monocytes and the NF-κB pathway in lipopolysaccharide (LPS)-stimulated RAW 264.7 macrophages. Additionally, chokeberry synergistically interacted with sodium selenite to inhibit NF-κB activation, cytokine release, and prostaglandin E_2_ (PGE_2_) synthesis [34]. Procyanidins C1, B2, and B5 in proanthocyanidin-rich fractions inhibited NO production in LPS-stimulated RAW 264.7 macrophages [35]. These data indicate that *Aronia melanocarpa* relieves the inflammation associated with various diseases.

**Table 1:** Role of *Aronia melanocarpa* as antioxidant and anti-inflammatory effects.

Type of Aronia Extracts (Key Component)	Disease	Cell or Animal Type	Stimulus (Intensity)	Working Conc. (Range) for Duration	Mode of Action	References
chokeberry juice	antioxidant	human	-	50 mL (4 weeks)	↓TBARS ↑SOD	[27]
*Aronia melanocarpa*	antioxidant	RAW 264.7	RANKL	50 ng/mL	↓NF- κB ↓c-Fos ↓NFATc1 ↓MAPKs	[28]
Aronia crude extract	antioxidant	aqueous humor of Lewis rats	LPS	100 μg each footpad	↓NO ↓PGE_2_ ↓TNF-α	[31]
	anti-inflammatory	RAW264.7	LPS	10 μg/mL	↓ iNOS ↓ COX-2	
*Aronia Melanocarpa* fruit extract	anti-inflammatory	HAECs	TNFα	10 μg/mL	↓ICAM-1 ↓VCAM-1 ↓NF-κB ↓ROS	[32]
Aronia bioactive fraction (ABF)	anti-inflammatory	BEAS-2B	LPS	1 μg/mL	↓TNF-α ↓IL-6,-8,-1β ↓CCL5 ↓COX-2, iNOS ↓ROS	[33]
*Aronia melanocarpa* (Michx.) Elliot concentrate (cyanidin-3-O-galactoside, caffeoylquinic acids)	anti-inflammatory	human peripheral monocytes RAW 264.7	LPS	10 ng/mL	↓TNF-α ↓IL-6, IL-8 ↓NF-kB ↓PGE_2_	[34]
*Aronia melanocarpa* (procyanidins C1, B5, B2)	anti-inflammatory	RAW 264.7	LPS	500 ng/mL	↓NO	[35]

Human bronchial epithelial cells (BEAS-2B), Cyclooxygenase-2 (COX-2), Human aortic endothelial cells (HAECs), Intercellular Adhesion Molecule-1 (ICAM-1), Inducible nitric oxide synthase (iNOS), Lipopolysaccharide (LPS), Mitogen-activated protein kinases (MAPKs), NO (Nitric Oxide), Prostaglandin E_2_ (PGE_2_), Reactive Oxygen Species (ROS), Superoxide dismutase (SOD), Thiobarbituric acid-reactive substances (TBARS), total cholesterol (TC), tumor necrosis factor-α (TNF-α), vascular cell adhesion molecule 1 (VCAM-1). “↑” increased; “↓” decreased.

### 3.3. Anti-Obesity Effects

Abnormal fat accumulation in overweight and obese individuals poses potential health risks [36]. These conditions are major risk factors for diabetes and cardiovascular diseases. Kim et al. demonstrated that treatment with *Aronia melanocarpa* extracts in high-fat diet (HFD)-induced obese mice led to notable decreases in body weight, serum triglyceride (TG), and low-density lipoprotein (LDL) cholesterol levels, along with improved insulin sensitivity compared to that in the controls [37]. They also revealed that fermented *Aronia melanocarpa* enhanced insulin sensitivity and alleviated weight gain induced by an HFD in male C57BL/6J mice. The fermented *Aronia melanocarpa*-treated group exhibited improved glucose tolerance and increased insulin sensitivity compared to the non-fermented group. The administration of *Aronia melanocarpa* polyphenol effectively decreased NO and pro-inflammatory cytokines (MCP-1, TNF-α, IL-1β, and IL-6) production in LPS-induced RAW264.7 cells while concurrently mitigating oxidative stress by adjusting glutathione peroxidase (GSH-Px), malondialdehyde (MDA), and catalase levels. This supplementation also improved obesity and glucose tolerance and decreased systemic inflammation in HFD-fed rats [38]. Wistar rats subjected to a high-fructose diet and then treated with *Aronia melanocarpa* extracts exhibited a reduction of epididymal fat, blood glucose, TG, cholesterol, and LDL-C levels. The extracts also increased plasma adiponectin levels and suppressed TNF-α and IL-6 [39].

*Aronia melanocarpa* displayed significantly reduced liver TG levels and notably lower expression levels of acetyl-CoA carboxylase, fatty acid synthase, and sterol regulatory element-binding protein in the liver of an HFD model than in the control group [40]. Another previous study examined the impact of 3-month supplementation with *Aronia melanocarpa*-based functional beverages on the lipid profile of aging rats. The aged control group exhibited age-related dyslipidemia characterized by reduced high-density lipoprotein (HDL)-C levels and elevated TG levels. The supplemented groups demonstrated a notable increase in HDL-C levels and decreased TC/HDL-C and LDL-C/HDL-C indices compared with the control group [41].

Phytochemical examination revealed that the primary components of the fermented *Aronia melanocarpa* showed a notable decline in cyanidin glycosides (3-glucoside and 3-xyloside) throughout the fermentation process [42]. Mice-fed Aronia berries containing phenolic phytochemicals, such as cyanidin 3-glycoside and chlorogenic acid, exhibited a significantly lower total visceral fat content than those supplemented with bilberry phytochemicals. Additionally, fasting blood glucose levels were significantly reduced in mice fed with Aronia phytochemicals for 4 weeks [43]. Significant body weight and food intake reductions were observed when C57BL/6N mice received daily oral doses of a cyanidin-3-O-galactoside-enriched extract from Aronia berries for 8 weeks. This treatment also resulted in a decline in serum leptin, insulin, TG, total cholesterol, and LDL cholesterol levels [44]. Daily intake of 500 mg of Aronia extract reduced TC and LDL-C levels in smokers. The cholesterol-lowering activity was most closely associated with the urinary levels of the methylated metabolites cyanidin-3-O-galactoside and peonidin-3-O-galactoside [45].

Extracts from *Aronia melanocarpa* berries, rich in anthocyanins, hindered NF-κB activation induced by LPS in RAW 264.7 cells and mouse bone marrow-derived macrophages. Supplementation with *Aronia melanocarpa* berry extract showed a trend toward decreased fasting serum glucose levels. This supplementation significantly inhibited the phosphorylation of NF-κB p65 in epididymal adipose tissue, accompanied by a decrease in the expression of Cd11b and TNF-α mRNAs in epididymal stromal vascular fraction cells [46] (Table 2).

### 3.4. Anti-Diabetic Effects

*Aronia melanocarpa* offers a promising option for managing diabetes as it effectively enhances glucose metabolism. In a Wistar rat model of type 2 diabetes mellitus (T2DM), daily oral administration of Aronia berry ethanol extract for 8 weeks significantly reduced blood glucose, serum insulin levels, and insulin resistance. Glucose tolerance and hepatic glycogen levels were also elevated [47]. Another study suggested that Aronia juice inhibited the increase in postprandial blood glucose levels by suppressing dipeptidyl peptidase IV, α-glucosidase, and angiotensin-converting enzymes, which play important roles in regulating carbohydrate metabolism and the onset of diabetes, respectively [48]. *Aronia melanocarpa* extract effectively ameliorated blood glucose levels and hyperinsulinemia in T2DM mice. In the liver of T2DM mice treated with this extract, a reduction was observed in ROS, IKKβ/NF-κB p65, and JAK2/Stat3/5B signaling pathways, leading to a decrease in the expression levels of iNOS, suppressor of cytokine signaling 3 (SOCS3), and inflammatory mediators. Furthermore, this extract-induced improvement in hyperinsulinemia resulted in the downregulation of SOCS3 by reducing phosphorylated-Stat5B in hepatocytes. This extract also promoted glucose uptake and metabolism while mitigating hepatocyte enlargement and inflammatory cell infiltration in the livers of T2DM mice [49]. In patients with T2D, the consumption of phenol-rich Aronia juice along with standard diabetic therapy resulted in improved health status. These findings suggest that Aronia juice is a promising agent for preventing and treating diabetes mellitus [50].

Aronia berries exhibited favorable effects in managing type 1 diabetes (T1D). T1D arises from an autoimmune response that damages the pancreatic β cells, leading to insufficient insulin production. Thus, T1D necessitates insulin supplementation and is associated with severe organ dysfunction owing to elevated blood glucose levels. Moreover, a previous study revealed that their antidiabetic effects, including those of the leaf extracts, are often observed in a streptozotocin-induced diabetic T1D model [51]. Oral administration of chokeberry juice for 6 weeks significantly reduced plasma glucose levels [51]. In the animal T1D model, Aronia berry ethanol extract administration reduced blood glucose levels and preserved pancreatic β cells. These findings suggest that Aronia berry consumption may offer protective effects for pancreatic β cells and could be a therapeutic approach for treating T1D [52].

Supplementation with 0.2% *Aronia melanocarpa* fruit extract reduced maltase and sucrase activities while increasing lactase activity in the small intestinal mucosa. Additionally, ingestion of the extract improved the antioxidant status, notably normalizing the concentration of a lipid peroxidation indicator (thiobarbituric acid-reactive substances) in organ tissues, such as the liver, kidney, and lung. Furthermore, cholesterol-lowering and distinct hypoglycemic effects were observed [53].

A previous clinical study investigated the impact of a 4-week supplementation regimen with Alixir 400 PROTECT^®^ (Standardized *Aronia L. mlanocarpa* Extract-SAE, Belgrade, Serbia) on clinical and biochemical parameters in individuals diagnosed with metabolic syndrome. Increased SAE intake is associated with favorable effects on body weight, TC, LDL, HDL, blood pressure, and glycemia [54]. Natural phenolic products regulate carbohydrate and lipid metabolism, blood sugar levels, and insulin resistance while mitigating oxidative stress and inflammation. Consequently, they have attracted significant attention for their potential in managing and preventing diabetes [55].

Aronia berries may exert anti-diabetic effects by alleviating hyperglycemia-induced oxidative stress (Table 2).

**Table 2:** Role of *Aronia melanocarpa* as anti-obesity and anti-diabetes.

Type of Aronia Extracts (Key Component)	Disease	Cell or Animal Type	Stimulus (Intensity)	Working Conc. (Range) for Duration	Mode of Action	References
*Aronia melanocarpa* (Amygdalin, prunasin)	Obesity	3T3-L1 cells	insulin (10 μg/mL)	10 μM	↓C/EBPα, PPARγ, ↓SREBP1c ↓TG, LDLC	[37]
		C57BL/6J mice	HFD	100, 200 mg/kg		
*Aronia melanocarpa* L.	Obesity	RAW264.7 cells	LPS	-	↓NO ↓TNF-α ↓IL-1β, IL-6 ↓MCP-1	[38]
Chokeberry extract (anthocyanins)	Obesity	Wistar rats	FRD	100, 200 mg/kg	↓TG, cholesterol, LDL ↓TNF-α, IL6	[39]
*Aronia melanocarpa* (AM)	Diabetes	Wistar rats	-	10 mL/kg (3 months)	↑HDL-C ↓LDL-C	[41]
*Aronia melanocarpa* (fermented)	Obesity	C57BL/6J mice	HFD	100 mg/kg	↓TG, FoxO1	[42]
*Aronia melanocarpa* E.	Diabetes	Male sprague-Dawley rats	-	300 mg/kg (every day for 4 weeks)	↓TG ↓hyperglycemia	[43]
*Aronia melanocarpa* extract (cyanidin-3-O-galactoside)	Obesity	C57BL/6N mice	HFD	50–200 mg/kg	↑HSL, β3-AR ↓C/EBPα, PPARγ, ↓SREBP1c	[44]
anthocyanin-rich aronia berry extract (ARN)	Obesity& inflammation	RAW 264.7	LPS	0.2%	↓ NF-κB ↓COX-2, iNOS ↓Glut1, G6pdh	[46]
*Aronia melanocarpa* berry extract (AMBE)	Diabetes	Wistar rats	STZ	100, 400 mg/kg	↑IRS-2, P13K ↑AKT, GSK-3β, GLUT2 ↓IL-1β, IL-6, TNF-α, ROS	[47]
Aronia juice	Obesity	C57BL/6JmsSlc KK-Ay mice	-	28 days	↓GIP, α-glucosidase ↓DPP IV	[48]
*Aronia melanocarpa* Anthocyanin Extracts	Diabetes	C57BL/6J male mice	STZ	150~300 mg/kg	↓SOCS3 ↓iNOS ↓JAK2/STAT3 ↑GLUT-4, GSK-3β	[49]
Aronia berry extract (70% ethanol)	Diabetes	ICR mice	STZ	10 mg/kg, 100 mg/kg	↓MAPK ↓NF-κB ↓COX-2, iNOS	[52]
chokeberry fruit extract	Diabetes	Wistar rats	STZ	20 mg/kg (4 weeks)	↓SOD ↓cholesterol	[53]

Beta 3 Adrenergic Receptor (B3-AR), Fructose-rich diet (FRD), High-fat diet(HFD), CCAAT Enhancer Binding Protein Alpha (CEBP-α), Dipeptidyl Peptidase-IV (DPP-IV), Forkhead box protein O1 (FOXO1), Glucose-dependent insulinotropic polypeptide (GIP), Hormone-sensitive lipase (HSL), Low-density lipoprotein (LDL), Peroxisome proliferator-activated receptor (PPAR), Reactive oxygen species (ROS), Sterol regulatory element binding protein-1c (SREBP1c), Triglycerides (TG), Tumor necrosis factor-α (TNF-α). “↑” increased; “↓” decreased.

### 3.5. Anti-Cancer Effects

Comparative analysis revealed that *Aronia melanocarpa* extract showed superior growth inhibition compared to the grape and bilberry anthocyanin-rich extracts [56]. A previous study suggested that *Aronia melanocarpa* juice selectively induced programmed cell death in T cell-derived lymphoblastic leukemia cells [57]. *Aronia melanocarpa* polyphenols regulated the expression of key regulators involved in G2/M cell cycle transition and apoptosis. Anticancer effects are primarily attributed to certain cyanidin glycosides, chlorogenic acids, and quercetin derivatives. The cyanidin glycosides found in Aronia berries inhibited the proliferation of HeLa cervical cancer cells and induced ROS production in these cells [58]. Phenolic compounds extracted from Aronia berries demonstrated significant antioxidant and cytotoxic effects against HepG2 liver cancer cells [59]. A previous study demonstrated that an anthocyanin-enriched blackberry extract exhibited antioxidant, anti-inflammatory, and antiproliferative properties against HT-29 cells [60]. Anthocyanin extracts resulted in 60% inhibition of human HT-29 colon cancer cell growth and exhibited cell cycle arrest at both the G1/G0 and G2/M phases [61]. The anthocyanins present in Aronia berries were also observed to suppress the growth of Caco-2 colon cancer cells via the Wnt/β-catenin signaling pathway [62]. Aronia berries exhibit promising potential against human colon cancer. Extracts from commercially available fruits of red (*Aronia arbutifolia* (L.) Pers.), purple (*Aronia prunifolia* (Marshall) Rehder), and black (*Aronia melanocarpa* (Michx.) Elliott) chokeberry species were evaluated for their total phenolic content, antioxidant activity, and growth inhibitory effects on HT-29 colon cancer cells. The results indicated that the Aronia berries displayed activity against HT-29 cells, which correlated with the total phenolic content, antioxidant activity, and concentrations of caffeic and chlorogenic acids [63]. Recently, two ester derivatives of ursolic acid, 3-O-trans- and -cis-p-coumaroyltormentic acids, suppressed the growth of MCF-7 and MDA-MB-231 breast cancer cells and inhibited mammosphere formation. This effect is achieved through the disruption of c-Myc expression, which is a crucial factor in cancer stem cell survival. These findings suggest that these triterpene esters exert potential inhibitory effects on breast cancer stem cells by targeting the c-Myc protein [64]. Another study demonstrated that exposure to Aronia juice inhibited the proliferation of Caco-2 cells [65]. They induce G2/M cell cycle arrest in Caco-2 cell growth by upregulating tumor suppressor carcinoembryonic antigen-related cell adhesion molecules. These findings suggest that Aronia berries and their components are promising candidates for developing novel anticancer agents (Table 3).

### 3.6. Cardioprotective Effects

Hypertension is a primary contributor to the development of cardiovascular and associated diseases and is often associated with endothelial dysfunction and oxidative stress [66,67,68]. Polyphenols potentially influence cardiovascular health and hypertension by mitigating oxidative vascular stress [69]. Aronia berries provide a rich reservoir of antioxidant compounds capable of boosting endothelial NO synthase activity, thereby reducing oxidative stress and expression levels of inflammatory genes [70]. The ethanolic extract of *Aronia melanocarpa* in the hypertension-induced animal model exhibited a decrease in blood pressure compared to the control group. Furthermore, the study highlighted that this reduction in blood pressure was associated with an enhancement in total antioxidant capacity and a decline in lipid peroxidation [70]. A previous study revealed that *Aronia melanocarpa* positively influenced blood pressure, NOS activity, and pro-inflammatory processes in N(ω)-nitro-L-arginine methyl ester (L-NAME)-induced hypertension [71]. A clinical study assessed the effects of Aronia berry juice on the transcriptome of peripheral blood mononuclear cells from 19 individuals at risk for cardiovascular disease. These findings suggest that prolonged habitual consumption of phenol-rich Aronia juice may induce immunomodulatory effects via various biological pathways [72]. Additionally, treatment with phenol-rich Aronia juice reduced the methylation levels of long interspersed nucleotide element-1 (LINE-1) and arachidonic acid/eicosapentaenoic acid ratio. Given the association between cardiovascular disease and alterations in DNA methylation, these findings suggest the potential cardioprotective effects associated with the habitual consumption of Aronia juice [73].

A meta-analysis of controlled clinical trials revealed that daily supplementation with Aronia berry extract for 6–8 weeks led to significant reductions in systolic blood pressure and total cholesterol levels among adult participants [74]. A previous study revealed that this supplementation with polyphenol-rich *Aronia melanocarpa* significantly downregulated the activity of glutathione-peroxidase (GSH-Px) in the hypertensive group, resulting in total antioxidant capacity values [75]. This administration significantly reduced blood pressure components and serum MDA compared to the hypertensive group. Endothelial progenitor cells (EPCs) treated with *Aronia melanocarpa* extract exhibited notable enhancements in proliferation and telomerase activity before exposure to angiotensin II. Conversely, the ratio of senescent cells and intracellular ROS formation decreased significantly compared to only angiotensin II-treated cells. Additionally, the extract augmented the migration ability and adhesion to fibronectin of EPCs while counteracting the angiogenic potential suppression induced by angiotensin II. These effects were attributed to the activation of the Nrf2 and the upregulation of HO-1 expression [76]. Administration of Aronia juice also significantly reduced the proatherogenic LDL fraction and contributed to a 16.5% decrease in total cholesterol levels in animals. The atherogenic indices of Aronia-supplemented animals exhibited a reduced atherogenic risk, while cardioprotective indices suggested safeguarding the cardiovascular system [77]. Aronia berry extract reduced TNF-α-induced monocyte/endothelial adhesion and suppressed vascular cell adhesion molecule-1 expression. Furthermore, the extract decreased the nuclear levels of both STAT3 and interferon regulatory transcription factor-1 [78].

A recent study reported that aged rats supplemented with *Aronia melanocarpa* fruit juice showed a significant decrease in the amount of collagen fibers and the expression level of the alpha-smooth muscle actin in their coronary tunica media, as compared with the old controls. Interestingly, the expression level of the angiotensin-converting enzyme 2 in the coronary tunica media of the supplemented group was significantly higher than in the control group, implying the positive effects of *Aronia melanocarpa* fruit juice supplementation on the age-dependent remodeling of coronary arteries [79].

Given the demonstrated link between oxidative stress and cardiovascular disease pathogenesis, the intake of dietary antioxidants should be encouraged for preventive measures. Phenolic compounds derived from berries target various signaling pathways crucial to the development of cardiovascular disease, including those involved in inflammation, oxidative stress, and cardiac and vascular remodeling. Since both estrogen and androgen receptors are distributed throughout the cardiovascular system, modulation of their activity can influence cardiovascular health and disease. Therefore, berry-derived phenolic compounds may offer a targeted approach to treating cardiovascular diseases [80,81]. Among these phenolic substances, chlorogenic acid and quercetin are potential lead compounds for the development of new agents in treating and preventing cardiovascular disease (Table 4).

### 3.7. Neuroprotective Effects

Several studies demonstrated that *Aronia melanocarpa* is effective in treating Alzheimer’s disease (AD) and improving cognitive decline with the aging process [82,83,84]. The administration of *Aronia melanocarpa* extracts markedly decreased NO production and mRNA levels of various inflammatory genes, including COX-2, IL-1β, iNOS, and TNF-α in LPS-induced BV2 cells, a type of microglial cell. Moreover, the results showed that *Aronia melanocarpa* extracts significantly mitigated tissue damage in the hippocampus of the mouse AD model. The neuroprotective effects of *Aronia melanocarpa extracts* and quinic acid against amyloid beta-induced cell death were confirmed in rat hippocampal primary neurons [82]. Another study demonstrated that pre-exposure to anthocyanins in *Aronia melanocarpa* significantly impeded Aβ1-42-induced apoptosis, reduced intracellular calcium and ROS levels, and enhanced ATP production and mitochondrial membrane potential. Anthocyanins upregulated the transcription of calmodulin and Bcl-2 genes while decreasing the expression of cytochrome c, caspase-9, cleaved caspase-3, and Bax proteins. Anthocyanins from *Aronia melanocarpa* shielded SH-SY5Y cells against Aβ1-42-induced apoptosis by modulating Ca^2+^ homeostasis and apoptosis-related genes, thus inhibiting mitochondrial dysfunction [83]. Lee HY et al. suggested that berries of *Aronia melanocarpa* exhibited a protective effect by attenuating glutamate-induced apoptosis in HT22 mouse hippocampal cells. These berries also lowered levels of ROS and intracellular Ca^2+^. Additionally, *Aronia melanocarpa* berries boosted glutathione levels, antioxidant enzyme activities (glutathione reductase and glutathione peroxidase), and mitochondrial membrane potential in HT22 cells [84].

Anthocyanin derived from *Aronia melanocarpa* fruit was found to inhibit age-related cognitive decline and diminished response capacity in senescence-accelerated mice. Additionally, mice supplemented with anthocyanins exhibited improved balance in redox systems (SOD, GSH-PX, and MDA). Levels of norepinephrine, dopamine, and 5-hydroxytryptamine were remarkably elevated, whereas levels of inflammatory cytokines (COX2, TGF-β1, and IL-1) and DNA damage were significantly reduced in the brains of anthocyanin-treated mice compared to the aged models [85]. Oral administration of cyanidin 3-O-β-galactoside derived from *Aronia melanocarpa* (Michx) Elliott effectively mitigated the decline in brain glucose uptake in aging mice. Additionally, cyanidin 3-O-β-galactoside alleviated neuronal damage in both the hippocampus and cortex regions. Specifically, the number of neurons increased in the hippocampus and the cortex. Moreover, cyanidin 3-O-β-galactoside reduced β-amyloid load in the brain and significantly improved performance in the Morris water maze test. Further investigation suggested that protein kinase B (AKT) might be the target of cyanidin 3-O-β-galactoside, contributing to its beneficial effects on brain energy metabolism [86]. Similarly, rats supplemented with *Aronia melanocarpa* (Michx) Elliot juice exhibited more vertical movements than aged controls. *Aronia melanocarpa* significantly enhanced the density of nerve fibers in the perforant path of the hippocampus and elevated acetylcholinesterase activity in the hippocampus, indicating improved functional activity of cholinergic neurons [87]. These studies imply that *Aronia melanocarpa* can help improve neuronal defects (Table 5).

### 3.8. Anti-Aging Effects

Glycation, also known as non-enzymatic glycosylation, refers to the covalent binding of a sugar molecule to a protein, lipid, or nucleic acid molecule [88,89]. This non-enzymatic process is accountable for numerous complications, such as micro and macrovascular issues, and is associated with certain diseases and skin aging [90,91].

The phenolic compounds found in *Aronia melanocarpa* exhibited a notable inhibitory impact on glycation products. Chlorogenic acid achieved 72.27% inhibition of fructosamine. Additionally, epigallocatechin gallate demonstrated 84.47% inhibition of α-dicarbonyl formation and 54.44% inhibition of advanced glycation end-products (AGEs) [92]. Cyanidin-3-O-galactoside, cyanidin-3-O-arabinoside, and procyanidin B2 also exhibited their potential as anti-glycation agents [93].

Several studies reported that *Aronia melanocarpa* has anti-aging effects in wrinkles, whitening, and moisture [94,95,96,97]. Treatment with the *Aronia melanocarpa* extract improved UVB-induced epidermal damage in mice. Furthermore, *Aronia melanocarpa* treatment significantly enhanced the expression level of collagen type I and III while downregulated MMP-1 and 3 compared to those in only UVB-exposed mice, meaning that *Aronia melanocarpa* extract mitigates UV-induced photodamage by attenuating UVB-induced collagen disruption. These effects may be owing to chlorogenic acid and rutin in the extract [94]. The Aronia extract promoted cell proliferation in neonatal human dermal fibroblasts. Treatment with the Aronia extract led to an increase in the transcription of collagen type I mRNA. Furthermore, the extract suppressed the expression of MMP1 and MMP3. In the dermal equivalents model, the compressive modulus, characterized by collagen synthesis, increased proportionally with the concentration of the Aronia extract, while expression levels of MMP1 and MMP3 decreased inversely with its concentration [95].

Pretreatment with *Aronia melanocarpa* concentrate downregulated the expression level of TNF-α-induced ICAM-I and subsequent monocyte adhesiveness in the HaCaT keratinocyte cells. Additionally, *Aronia melanocarpa* concentrate significantly diminished intracellular ROS generation and mitigated mitogen-activated protein kinase activation in TNF-α-induced HaCaT cells. Both *Aronia melanocarpa* concentrate and its component cyanidin 3-glucoside also alleviated TNF-α-induced IKK activation, IκB degradation, the nuclear translocation of p65, and p65 DNA binding activity in HaCaT cells [96]. Our group also investigated how fermented *Aronia melanocarpa* (FA), fermented with *Monascus purpureus*, inhibits melanogenesis and its underlying mechanism in the B16F10 melanoma cell line. FA effectively suppressed tyrosinase activity and melanogenesis in alpha-melanocyte-stimulating hormone-induced B16F10 cells. Notably, FA downregulated the PKA/CREB pathway, reducing protein levels of tyrosinase, TRP-1, and MITF. Additionally, FA inhibited MITF transcription by enhancing the phosphorylation levels of both GSK3β and AKT. Interestingly, these effects were attributed to a significant upregulation in gallic acid, a compound of *Aronia melanocarpa* generated following the fermentation process with *Monascus purpureus* [97].

These data suggest that *Aronia melanocarpa* can be utilized as a cosmetic ingredient for anti-aging skin (Table 6).

## 4. Conclusions

This review covers the latest research trends on the positive effects of *Aronia melanocarpa* on a variety of diseases, including diabetes, cardiovascular disease, and neurological diseases. Many studies have reported the specific effects of anthocyanins and phenolic acids, the main components of *Aronia melanocarpa*. However, polyphenol monomers and components with potent functional and disease-controlling properties in *Aronia melanocarpa* should be systematically identified. While phenolic compounds are recognized as the primary active components of Aronia berries, the specific anti-diabetic, anti-obesity, and neuroprotective agents within these berries have yet to be clearly identified. Further research is also required to investigate ursolic acid, its derivatives, and other constituents of Aronia berries that demonstrate potential anti-tumor activity. Additionally, future research on *Aronia melanocarpa* should focus on optimizing the doses of phenolic and other constituents, developing new formulations, isolating new active compounds, and exploring their synthetic modifications.

Although much research revealed their beneficial effects on preventing and treating diseases linked to oxidative stress, clinical trials have shown limited effectiveness so far. Thus, clinical studies based on a better understanding of the main components of *Aronia melanocarpa* are needed in the future. These future studies will contribute to using *Aronia melanocarpa* as a resource for a therapeutic agent to delay or prevent various diseases.

## Figures and Tables

**Table 3:** Role of *Aronia melanocarpa* and its underlying mechanism for improving cancer.

Type of Aronia Extracts (Key Component)	Disease	Cell or Animal Type	Stimulus (Intensity)	Working Conc. (Range) for Duration	Mode of Action	References
*Aronia melanocarpa* juice (AMJ)	colon/colorectal cancer	Jurkat cell	MnTMPyP PEG-SOD PEG-Catalase	0.3 *v*/*v*	↑ROS, Cytochrome c ↓P73, Cleaved caspase 3 ↑UHRF1	[57]
Aronia berries (cyanidin glycosides)	cervical cancer	HeLa	-	0~200 μg/mL	↑ROS	[58]
Chokeberry (Phenolic compound)	liver cancer	HepG2	-	0~600 μg/mL	↓ROS	[59]
Hull blackberry extract (HBE) (anthocyanin)	colorectal cancer	HT-29	-	5.1~37.3 μM	↓IL-12	[60]
Anthocyanin-Rich Extract	colon cancer	HT-29	-	50 μg/mL	↓COX-1 ↑COX-2, PGE2	[61]
*Aronia melanocarpa* Elliot anthocyanins (anthocyanins)	colorectal cancer	Caco-2	-	50–200 μg/mL	↓SOD, BAX, Cleaved Caspase-3, GSK3β, β-Catenin ↑MDA, E-cadherin	[62]
*Aronia melanocarpa* juice (AMJ) (3-O-P-Coumaroyltormentic Acid)	breast cancer	CSCs	radiation	20 μM	↓Sox2, Oct4, CD44 ↓C-Myc	[64]
*Aronia melanocarpa* juice	Colon cancer	Caco-2	-	5% for 4 days	↑G(2)/M cell cycle arrest ↓CEACAM1	[65]

AMP-dependent kinase (AMPK), Construction skills certificate scheme (CSCs), Colon adenocarcinoma (Caco-2), Cyclooxygenase 1, 2 (COX-1, 2), Henrietta Lacks (HeLa), Human hepatocellular carcinoma (HepG2), Malondialdehyde (MDA), mammalian target of rapamycin complex 1 (mTORC1), Prostaglandin E2 (PGE2), Sprague-Dawley (SD) Rat, Superoxide dismutase (SOD), tumor suppressor carcinoembryonic antigen-related cell adhesion molecule 1 (CEACAM1). “↑” increased; “↓” decreased.

**Table 4:** Role of *Aronia melanocarpa* and its underlying mechanism for improving cardiovascular diseases.

Type of Aronia Extracts (Key Component)	Disease	Cell/Animal Type	Stimulus (Intensity)	Working Conc. (Range) for Duration	Mode of Action	References
*Aronia melanocarpa*	Hypertension	WKY rat	L-NAME 40 mg/kg/day	57.90 mg/kg/day for 3 weeks	↑eNOS, ↓IL-6 ↓TNF-α	[71]
*Aronia melanocarpa* juice	CVDs	PBMC	-	-	↓TNF-α ↓DUSP2 ↓IL-8,-1β	[72]
*Aronia melanocarpa* juice (AMJ)	Dyslipidemia	venous blood	-	100 mL (everyday for 4 weeks)	↓LINE-1 methylation ↓AA/EPA levels	[73]
*Aronia melanocarpa* Elliot	arterial hypertension	Wistar white rats	L-NAME	40 mg/kg (every 2 days, for 8 weeks)	↓ROS ↑SOD ↑CAT	[75]
*Aronia melanocarpa* fruit extract	atherosclerosis myocardial infarction	EPCs	angiotensin II	1–25 μg/mL	↓ROS ↑Nrf2 ↑HO-1	[76]
Aronia melanocarpa Fruit Juice	atherosclerosis	Wistar rats	-	64 mL/kg	↓TC ↓ROS, LDL-C	[77]
Aronia berry extract	atherosclerosis	HUVECs	TNF-α	10 ng/mL	↓ IL-6,-8,-1β ↓MCP-1 ↓VCAM-1 ↓IRF1 ↓gp130, STAT3, ↓IL-1β, IL-6, IL-6ST ↓CXCL8, CCL2	[78]
Aronia melanocarpa fruit juice (AMJ)	coronary arteries	Wistar rats	-	-	↓α-SMA ↑ACE2	[79]

Arachidonic acid (AA), Angiotensin-converting enzyme (ACE2), Catalase (CAT), Cyclooxygenase-2 (COX-2), premature cardiovascular diseases (CVDs), Dual Specificity Phosphatase 2 (DUSP2), eicosapentaenoic acid (EPA), Endothelial progenitor cells (EPC), Human aortic endothelial cells (HAECs), High Density Lipoprotein-cholesterol (HDL-C), Heme oxygenase 1 (HO-1), human umbilical vein endothelial cells (HUVECs), Interferon regulatory factor 1 (IRF1), Low-density lipoprotein- cholesterol (LDL-C), N(ω)-nitro-L-arginine methyl ester (L-NAME), MCP-1 (Monocyte chemoattractant protein-1), NFATC1 (Nuclear Factor Of Activated T Cells 1), Nuclear factor erythroid-2-related factor 2 (Nrf2), Peripheral blood mononuclear cells (PBMC), Reactive Oxygen Species (ROS), Superoxide dismutase (SOD), total cholesterol level (TC), tumor necrosis factor-α (TNF-α), vascular cell adhesion molecule 1 (VCAM-1). “↑” increased; “↓” decreased.

**Table 5:** Role of *Aronia melanocarpa* and its underlying mechanism for improving neuronal diseases.

Type of Aronia Extracts (Key Component)	Disease	Cell or Animal Type	Stimulus (Intensity)	Working Conc. (Range) for Duration	Mode of Action	References
Black chokeberry fruit (BCE)	Alzheimer’s	BV2	LPS	30–1000 μg/mL	↓iNOS, COX2, IL-1β, TNF-α	[82]
		ICR mice	LPS	50 mg/kg		
Anthocyanins from Black Chokeberry	Alzheimer’s	SH-SY5Y	Amyloid beta peptide (1–42) Aβ1-42	20–60 mg/mL	↓Caspase9, Bax, cytochrome C ↑Bcl-2, Calmodulin	[83]
*Aronia melanocarpa* berries	neurodegenerative	HT22	Glutamate	10–100 μg/mL	↓ROS ↑GSH, GPx, GR	[84]
Anthocyanins from Black Chokeberry	neurodegenerative	Kunming mice	D-galactose	15–30 mg/kg	↓COX2, TGF-β1, IL-1, ROS, ATM, ATR	[85]

Ataxia telangiectasia mutated (*ATM*), Ataxia telangiectasia and Rad3-related protein (*ATR*), Cyclooxygenase (COX-2), Glutathione (GSH), Glutathione peroxides (*GPx*), glutathione reductase (*GR*), Inducible nitric oxidase (iNOS), Reactive Oxygen Species (ROS), Tumor necrosis factor (TNF-α), thrice cloned subline of the neuroblastoma cell line SK-N-SH (SH-SY5Y). “↑” increased; “↓” decreased.

**Table 6:** Role of *Aronia melanocarpa* and its underlying mechanism for improving aging.

Type of Aronia Extracts (Key Component)	Disease	Cell or Animal Type	Stimulus (Intensity)	Working Conc. (Range) for Duration	Mode of Action	References
*Aronia melanocarpa* extract (chlorogenic acid, rutin)	Anti-aging	ICR mice	UVB (150 mJ/cm)	1%/1 week	↑collagen I/III ↓MMP-1, MMP-3	[94]
Aronia extract	Anti-aging	HDFs	NONE	1–100 μg/mL	↓MMP-1, MMP-3 ↑collagen I	[95]
*Aronia melanocarpa* concentrate (cyanidin 3-glucoside)	Anti-melano-genesis	HaCaT	TNF-α	10 ng/mL	↓ROS ↓MAPKs	[96]
Fermented *Aronia melanocarpa* (gallic acid)	Anti-melano-genesis	B16F10	α-MSH (500 nM)	500 μg/mL	↓PI3K/AKT ↓PKA/CREB	[97]

Institute of cancer research (ICR), High-fat diet (HFD), α-Melanocyte-stimulating hormone (α-MSH), Mitogen-activated protein kinases (MAPKs), Human dermal fibroblasts (HDFs), Human keratinocytes cells (HaCaT), InterLeukin-6 (IL-6), Matrix Metalloproteinase-1 (MMP-1), Matrix Metalloproteinase-3 (MMP-3), Phosphoinositide 3-kinases (PI3Ks), Protein kinase A (PKA), cAMP-responsive element binding protein (CREB), Reactive oxygen species (ROS), Tumor necrosis factor-α (TNF-α). “↑” increased; “↓” decreased.
